# Pain Treatment in Primary Care Through Eight Constitution Medicine: A Retrospective Real-World Study from South Korea

**DOI:** 10.3390/medicina61091564

**Published:** 2025-08-30

**Authors:** Nahyun Cho, Younkuk Choi, Heekyung Kim, Jeongmi Yun, Hyungsun Jun, Changsop Yang, Sungha Kim, Jungtae Leem

**Affiliations:** 1 Department of Diagnostics, College of Korean Medicine, Wonkwang University, Iksan 54538, Republic of Korea; jnh528@naver.com; 2 East–West Cancer Center, Cheonan Korean Medical Hospital, Daejeon University, Cheonan 34520, Republic of Korea; 3 Department of Clinical Research Design and Evaluation, Samsung Advanced Institute for Health Sciences & Technology (SAIHST), Sungkyunkwan University, Gangnam-gu, Seoul 06355, Republic of Korea; pyrologos@gmail.com (Y.C.); yebon8@gmail.com (H.K.); 4 Gangnam-Shingwang ECM Clinic, Seocho-gu, Seoul 06612, Republic of Korea; 5 Yebon ECM Clinic, Songpa-gu, Seoul 05510, Republic of Korea; 6 Yeson ECM Clinic, Yangcheon-gu, Seoul 07999, Republic of Korea; yeson1004@naver.com; 7 Department of Diagnostics, College of Korean Medicine, Dongshin University, Naju 58245, Republic of Korea; hs14231423@naver.com; 8 KM Science Research Division, Korea Institute of Oriental Medicine, Daejeon 34054, Republic of Korea; yangunja@kiom.re.kr; 9 Clinical Medicine Division, Korea Institute of Oriental Medicine, Daejeon 34054, Republic of Korea; 10 Research Center of Traditional Korean Medicine, College of Korean Medicine, Wonkwang University, Iksan 54538, Republic of Korea; 11 Department of Il-won Integrated Medicine, Wonkwang University Korean Medicine Hospital, 895, Muwang-ro, Iksan 54538, Republic of Korea

**Keywords:** acupuncture, precision medicine, musculoskeletal pain, healthy lifestyle, retrospective studies

## Abstract

*Background and Objectives*: Musculoskeletal pain is a global public health issue. Eight Constitution Medicine (ECM), a type of East Asian Traditional Medicine, offers personalized, minimally invasive treatment through Eight Constitution Acupuncture (ECA) and Eight Constitution Lifestyle Intervention (ECLI). Despite its clinical use, scientific evidence supporting ECM’s effectiveness remains limited. This study aimed to evaluate the effectiveness in treating musculoskeletal pain in primary care settings. *Materials and Methods*: This retrospective study analyzed medical records from three ECM clinics (Gangnam-Shingwang, Yeson, and Yebon) between January 2018 and August 2023. A total of 163 patients were included, with 44 providing follow-up data. Pain intensity, quality of life, and functional outcomes were assessed using validated instruments including the PainDETECT questionnaire, Korean Cancer Pain Assessment Tool (KCPAT) somatic pain scores, EuroQol 5-Dimension 5-Level (EQ-5D-5L), Western Ontario and McMaster Universities Osteoarthritis Index (WOMAC), Oswestry Disability Index (ODI), Neck Disability Index (NDI), and Shoulder Pain and Disability Index (SPADI). Pre- and post-treatment scores were statistically analyzed. *Results*: Significant decreases were observed in KCPAT somatic pain scores (11.77 ± 4.77 to 9.77 ± 5.32) and significant improvements in EQ-5D-5L scores (0.74 ± 0.12 to 0.80 ± 0.07). WOMAC and ODI scores also showed significant improvements. However, the changes in the NDI, SPADI, and PainDETECT scores were not statistically significant. No adverse events were reported. *Conclusions*: ECM, through ECA and ECLI, may offer effective personalized treatment for musculoskeletal pain, improving both pain intensity and quality of life. Despite its small sample size and retrospective design, this study offers valuable preliminary evidence for ECM. Further large-scale prospective studies are needed to confirm these findings.

## 1. Introduction

Musculoskeletal pain is the most frequently reported medical condition worldwide. The age-standardized prevalence rate of musculoskeletal pain per 100,000 people globally is 16,276.2 [1], and in South Korea, it is 11,212.7 per 100,000 people, accounting for the highest proportion (15.3%) of the country’s total disability-adjusted life years (DALYs) [2]. South Korea is among the fastest aging nations in the world, and the prevalence of musculoskeletal disorders has increased by 26.8% from 2010 to 2018 [3]. The burden of musculoskeletal diseases on the overall healthcare system, both medically and economically, is significant and is expected to increase as the population continues to age [4].

Non-steroidal Anti-Inflammatory Drugs and acetaminophen are the most prescribed drugs for musculoskeletal pain. However, these conventional medications are not recommended for long-term use because of their significant side effects such as weight gain, weight loss, gastrointestinal symptoms, and dizziness [5]. As patient satisfaction with standard treatments decreases, there is growing demand for alternative integrative medical approaches [6]. Recent studies have suggested that acupuncture can be effective in managing pain and improving function in patients with low back pain (LBP) [7], neck [8], shoulder [9], and knee pain [10], indicating its increasing use in pain management. In South Korea, where acupuncture is fully covered by national health insurance, approximately 40% of primary care visits to Traditional Korean Medicine(TKM) practitioners are for musculoskeletal disorders, indicating a high utilization rate within this specialty when considering the total number of primary TKM clinics [3]. East Asian Traditional Medicine (EATM), which includes TKM, employs a holistic framework that goes beyond simple acupuncture to systematically links psychosomatic and lifestyle factors for diagnosis and treatment. Historically and culturally, acupuncture has been associated with healthy lifestyle habits [11]. Furthermore, lifestyle intervention, including diet control, sleep, and exercise, has been traditionally emphasized in EATM. Previous studies have shown that managing musculoskeletal pain requires not only medical treatment but also lifestyle modifications [12]. Although some studies have explored acupuncture combined with exercise or dietary interventions [13,14], there is still a lack of research assessing their comprehensive, individualized integration within structured models, such as constitution-based medicine in primary care setting. This gap highlights the need for studies that evaluate the systematic linkage of psychosomatic and lifestyle factors for diagnosis and treatment.

In South Korea, eight constitution medicine (ECM) combines acupuncture treatment and lifestyle intervention. It is a unique constitutional medicine system in TKM. Its diagnostic approach involves dividing individuals into eight inherent constitutions based on radial artery pulse patterns identified through pulse diagnosis [15]. In ECM, the minimally invasive Eight Constitution Acupuncture (ECA) and personalized Eight Constitution Lifestyle Intervention (ECLI), which include diet and exercise tailored to each constitution, are comprehensively used for treatment [16]. However, although it is widely used in clinical practice owing to its observed effectiveness, it lacks sufficient clinical research evidence [17,18,19]. Additionally, there is currently no clinical research on the effect of ECLI on musculoskeletal pain. However, an increasing number of studies have recently begun to report supporting evidence [20].

This multi-institutional retrospective observational study aimed to evaluate the real-world clinical effectiveness of combined ECA and ECLI therapy for musculoskeletal pain. Furthermore, we sought to determine the impact of treatment on patients’ quality of life and to understand the factors contributing to diverse treatment responses and follow-up patterns. This included identifying characteristics that may influence patient outcomes and the overall effectiveness of this therapeutic approach.

## 2. Materials and Methods

We analyzed the medical records of patients who visited the ECM clinics for back, neck, shoulder, and knee pain. We observed the effects of ECA and ECLI by evaluating symptoms before and after treatment. This was a single-group retrospective chart review study without a control group and was preliminary in nature. All authors who designed and conducted this study are licensed Korean Medicine Doctors (KMDs). Authors Y.C., H.K. and J.Y. have direct clinical experience in ECM and provided the patient data for this study. The remaining co-authors (N.C., H.J., C.Y., S.K., and J.L.) are clinical research experts and specialists in traditional medicine. The other co-authors, although without direct ECM clinical experience, contributed their expertise in research methodology and have published relevant academic papers. The Wonkwang University Bioethics Committee approved the study protocol (IRB no: WKIRB-202308-BM-061) on 9 August 2023. Because this was a retrospective study, the need for prior consent was waived by the IRB.

### 2.1. Participants

This retrospective study used data from patients who visited the Gangnam-Shingwang, Yeson, and Yebon clinics between January 2018 and August 2023, and reported one of the following symptoms: neck, shoulder, back, or knee pain. The diagnostic codes for each pain site were as follows: for neck pain, “Pain in joint, other (M25.58)” or “Cervicalgia, cervical region (M54.22)”; for shoulder pain, “Pain in joint, shoulder region (M25.51)”; for low back pain, “Low back pain, lumbar region (M54.56)”; and for knee pain, “Pain in joint, lower leg (M25.56)”. Patients in this study were diagnosed with nonspecific musculoskeletal conditions, such as myofascial pain syndrome. Detailed differential diagnosis into specific anatomical pathologies like osteoarthritis or tendinopathy was not the primary focus of diagnosis, as the therapeutic approach in EATM focuses on the patient’s symptoms and constitutional type, rather than the specific anatomical structure involved.

Patients aged between 20 and 80 years were included in this study, without restrictions on sex, comorbidities, symptom severity, or disease duration, encompassing all individuals who visited the clinics during the study period. Patients whose records did not confirm the interventions were excluded from this study. In addition, patients with suspected serious conditions (e.g., fractures, infections, or malignancies), identified through clinical assessments by the attending KMDs and referred to conventional medical institutions, were also excluded.

### 2.2. Intervention

Eligible participants underwent standard treatment processes used in clinical practice at the Gangnam-Shingwang, Yeson, and Yebon clinics in Seoul. The treatments, ECA and ECLI, were performed by authors Y.C., H.K., and J.Y., who are licensed KMDs with about 20 years of clinical experience. KMDs are practitioners of TKM within a dual healthcare system of South Korea. All have obtained a master’s degree or higher in Clinical Epidemiology or related fields. The first step involved classifying the patients’ constitutional groups using a standardized pulse diagnosis technique [15,21].

**Figure 1:**
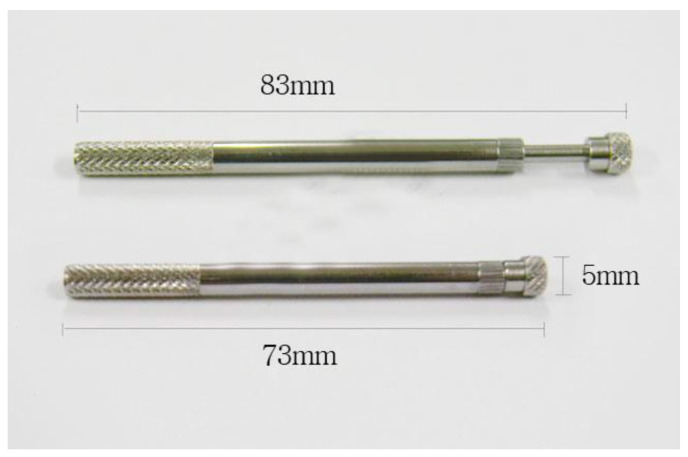
Standard ECA guide tube.

Once the constitution group was determined, acupuncture was administered at different acupoints depending on the symptoms [16]. The fast-in, fast-out method of this acupuncture was used, employing standard ECA guide tube (Figure 1). The standard frequency of ECA treatment was three times per week for three months, adjustable based on the clinician’s judgment. Each treatment session lasted <20 min [22].

Because this study reflects real-world clinical protocols used in primary care settings, there were no strict restrictions on concurrent treatments. During this treatment period, the use of herbal medicine was generally restricted, and the use of opioid analgesics was avoided. However, the concurrent use of non-opioid analgesics was permitted. These restrictions were flexibly applied based on the patient’s condition and clinical discretion.

The patients were informed about ECLI, which included diet, bathing, and exercise methods specific to each constitution [16]. The diet varied for each constitution, with different recommendations for seafood, meat, vegetables, and fruits. The recommended exercises and bathing methods (hot or cold baths) also varied according to constitution [23]. The ECLI plan was provided in paper format, with instructions provided by a physician or a trained research assistant (Supplementary File S1).

### 2.3. Outcome Measurements and Data Collection

This study evaluated the general symptom scores for pain, symptom-specific indices for each body part, and quality of life to assess the effects of ECA and ECLI. Patient-Reported Outcomes (PROs) were used, considering the standardization of outcome measures and feasibility in the clinical setting. These outcomes were collected using both paper and electronic surveys, and their completion was recorded in electronic medical records. These surveys are part of the standard clinical protocols of the three primary care institutions.

Baseline information included sex, date of birth, educational background, marital status, job, and one of eight constitutional diagnoses determined by the physician. General information related to pain, such as alcohol consumption and smoking history, onset, cause, aggravating factors, diagnosis, medical and surgical history, current illness, and medication history, was also collected.

The primary outcome was the change in symptom-specific indexes (Neck Disability Index [NDI], Oswestry Disability Index [ODI], Western Ontario and McMaster Universities Osteoarthritis Index [WOMAC], and Shoulder Pain and Disability Index [SPADI]) from the first visit (Visit 1) to follow-up. Secondary outcomes included changes, in general, symptom scores (PainDETECT score), somatic pain scores from the Korean Cancer Pain Assessment Tool (KCPAT), and EuroQol 5-Dimension 5-Level (EQ-5D-5L) scores from visit one to follow-up.

The PRO surveys were collected at the first visit and 4–8 weeks later at follow-up, and the data were stored in a database. Data meeting the selection and exclusion criteria were extracted in Excel.

The NDI is a tool used to assess neck and cervical spine dysfunction and comprises 10 items scored from 0 to 5, with higher scores indicating more severe activity impairment [24,25]. The minimal clinically important difference (MCID) for the nonspecific neck pain NDI score was set at 3.5 points in this study based on previous results [26]. ODI, which is used to evaluate functional disability due to LBP, consists of 10 items with the MCID set at 10 points [27,28]. WOMAC score, designed to assess knee joint function, includes five items for pain, two for stiffness, and 17 for physical function, scored on a five-point scale, with higher scores indicating a worse knee joint condition [29,30]. The MCID was set at 7.9 points [31]. SPADI assesses shoulder pain and disability using five pain items and eight functional activity items, and is scored 0–10, with higher scores indicating more severe disability [32,33]. The MCID was set to 8 points [34].

PainDETECT is a scale consisting of seven items for assessing neuropathic pain, with scores ranging from −1 to 38, where higher scores indicate more severe neuropathic pain [35,36]. KCPAT, developed for the initial assessment of cancer pain in Korean adults, collects data on six items of somatic pain from the pain character average intensity score, and is scored between 6 and 30 points, with higher scores indicating more severe pain [37].

The Korean version of the EQ-5D-5L was used to assess the health-related quality of life. It consists of five domains: mobility, self-care, usual activities, pain/discomfort, and anxiety/depression. Each domain is scored from 1 to 5, with lower scores indicating a healthier state [38,39]. The MCID for EQ-5D-5L was set at 0.18 points [40].

### 2.4. Statistical Analysis

#### 2.4.1. Descriptive Statistics Summary of Study Participants

Descriptive analysis was performed for the collected baseline characteristics. For continuous variables, mean ± standard deviation (SD), median, and interquartile range are presented, and for categorical variables, frequency and ratio are provided.

#### 2.4.2. Clinical Response Evaluation Criteria and Methods

Clinical responses were evaluated using paired *t*-test analysis (or Wilcoxon signed-rank test, if not normally distributed) for changes in continuous variables (symptom-specific, pain, and quality of life scores) from baseline to follow-up. The effect size was calculated using standardized mean difference.

The responder and non-responder groups were determined based on the MCID, and the proportion of responders in each symptom group and overall was investigated. Differences in characteristics between these groups were analyzed using the chi-squared test (or Fisher’s exact test) for nominal variables and the independent *t*-test (or Wilcoxon rank-sum test) for continuous variables.

#### 2.4.3. Factors Influencing Treatment Effectiveness

To identify factors influencing treatment effectiveness, we performed univariate linear regression analysis for continuous dependent variables (SPADI, WOMAC, NDI, ODI, and EQ-5D-5L scores) and univariate logistic regression analysis for the binary dependent variable (treatment responders and non-responders based on MCID). Covariates included sex, age, diagnostic constitution, education, marital status, job, smoking history, drinking history, supplementary diet intake, baseline PainDETECT, KCPAT somatic pain scores, and EQ-5D-5L scores.

Covariates with statistical significance in univariate analysis were then included in a multivariate linear/logistic regression analysis to identify independent factors influencing treatment effectiveness.

All statistical analyses were performed using R Version 4.3.2 software (R Studio, Boston, MA, USA). Statistical significance was set at *p* < 0.05.

#### 2.4.4. Safety Evaluation Criteria

The safety evaluation involved reviewing all adverse events recorded in the charts during the treatment period. Adverse event data were collected from the patient reports and clinician observations at each visit, and the incidence rates of adverse reactions, including those leading to discontinuation of treatment due to adverse reactions and serious adverse reactions, were reported.

## 3. Results

### 3.1. General Characteristics of Patients

**Figure 2:**
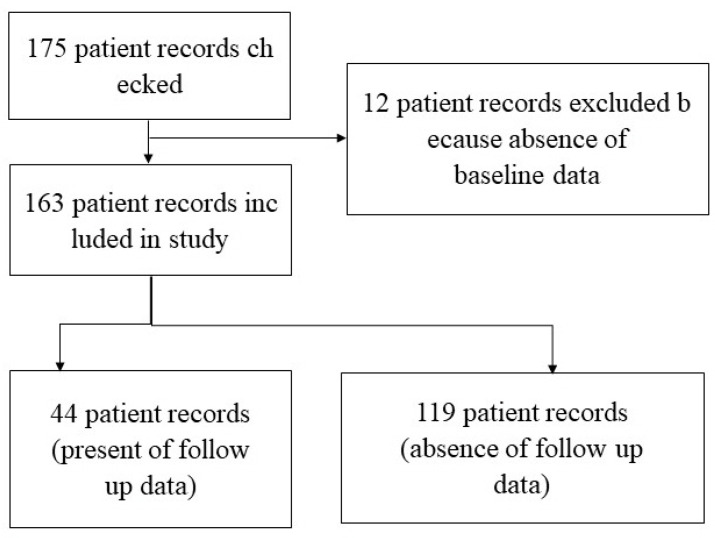
A Flowchart Depicting Data Extraction.

Data from 163 patients were analyzed: 26 with knee pain, 49 with LBP, 62 with neck pain, and 26 with shoulder pain (Figure 2). The average age of the 163 participants was 49.9 ± 14.3 years, with women comprising 72.4% (118 patients). The most common complaint was neck pain (62 patients, 38.0%), followed by LBP (49 patients, 30.1%). Knee and shoulder pain occurred in 26 patients each (16.0%). The most common constitutional diagnosis was pancreotonia (36 patients, 22.1%), followed by hepatonia (34 patients, 20.9%), colonotonia (29 patients, 17.8%), and others. The average baseline scores for PainDETECT, KCPAT somatic pain, and EQ-5D-5L were 9.2 ± 5.4, 11.7 ± 5.8, and 0.7 ± 0.1, respectively (Table 1). Age distribution by pain type showed knee pain at 55.0 ± 14.1 years, LBP at 50.7 ± 16.3 years, neck pain at 45.4 ± 13.0 years, and shoulder pain at 53.8 ± 10.1 years (Table S1).

Among the 163 patients, 44 had successful follow-up, with a significant age difference between the successful (53.91 ± 11.40 years) and unsuccessful follow-up groups (48.39 ± 14.97 years) (*p* < 0.05). A statistically significant difference was also noted in the ODI scores of patients with LBP between the successful (30 individuals, 155.38 ± 51.69 points) and unsuccessful (19 individuals, 122.70 ± 53.33 points) follow-up groups (*p* < 0.05). No other significant differences were observed between the groups (Table S2). Among the 44 individuals with successful follow-up, there were no statistically significant differences in the basic characteristics across the four pain subgroups, nor among the 119 individuals with unsuccessful follow-up, except for age (*p* < 0.05) (Table S3).

### 3.2. Clinical Response

#### 3.2.1. Pre- and Post-Treatment Comparison

For the 44 participants who completed the ECA and ECLI and had successful follow-ups, the Shapiro–Wilk normality test assumed a normal distribution for outcome variables such as PainDETECT, partial KCPAT, EQ-5D-5L, WOMAC, ODI, NDI, and SPADI scores.

Paired *t*-test analysis of pre- and post-treatment scores showed a significant decrease in partial KCPAT scores from 11.77 ± 4.77 to 9.77 ± 5.32 (*p* < 0.05) and a significant increase in EQ-5D-5L scores from 0.74 ± 0.12 to 0.80 ± 0.07 (*p* < 0.05). WOMAC scores decreased from 46.13 ± 18.08 to 31.50 ± 20.24 (*p* < 0.05) and ODI scores decreased from 155.38 ± 51.69 to 122.57 ± 79.40 (*p* < 0.05). However, the changes in the NDI and SPADI scores were not statistically significant after treatment.

**Figure 3:**
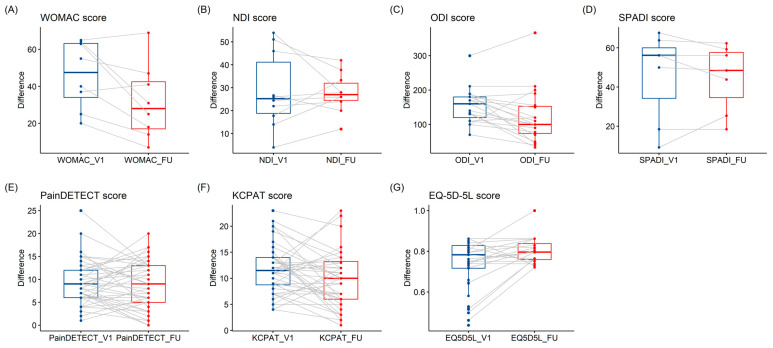
Comparison of Scores Before and After Treatment. (**A**) Comparison of WOMAC Scores (n = 8). (**B**) Comparison of NDI Scores (n = 10). (**C**) Comparison of ODI Scores (n = 19). (**D**) Comparison of SPADI Scores (n = 7). (**E**) Comparison of PainDETECT Scores (n = 44). (**F**) Comparison of KCPAT Scores (n = 44). Only the somatic pain item scores from the KCPAT were utilized. (**G**) Comparison of EQ-5D-5L Scores (n = 20). EQ-5D-5L = EuroQol-5 dimension-5L; FU = follow-up; KCPAT = Korean Cancer Pain Assessment Tool; NDI = Neck Disability Index; ODI = Oswestry Disability Index; SPADI = Shoulder Pain and disability Index; WOMAC = Western Ontario and McMaster Universities Osteoarthritis Index, V1 = baseline.

Overall PainDETECT scores and those for LBP specifically decreased from 9.32 ± 4.76 to 8.93 ± 5.06 and from 8.79 ± 3.43 to 6.89 ± 5.03, respectively; however, these changes were not statistically significant. PainDETECT scores for knee, neck, and shoulder pain increased post-treatment; however, these were not statistically significant. Neck pain partial KCPAT scores decreased from 14.30 ± 4.47 to 13.50 ± 4.70; however, this was not statistically significant; shoulder pain partial KCPAT scores increased post-treatment, but not significantly (Table 2, Figure 3).

Although PainDETECT scores decreased in all age groups except those in their 50s and those aged 70 and above, the changes were not statistically significant. In the 50s and 70+ age groups, PainDETECT scores increased, but these changes were also not statistically significant. Additionally, KCPAT scores remained the same or decreased across all age groups, yet none of the changes reached statistical significance (Table S4).

#### 3.2.2. Comparison Between Responder and Non-Responder Groups

Among the ten patients with neck pain, four (40%) were NDI treatment responders. Of the 19 patients with LBP, 14 (73.68%) were ODI responders. Among the shoulder pain patients, two of seven (28.57%) were SPADI responders, and six of eight knee pain patients (75%) were WOMAC responders. Overall, 26 of the 44 individuals (59.0%) were responders across various symptom-specific scores. Responders took dietary supplements more frequently and had significantly lower baseline EQ-5D-5L scores than non-responders. No differences were observed in the other variables (Table S5). Univariate logistic regression analysis of all covariates in the responder and non-responder groups did not yield statistically significant results (Table S6).

Of the 20 individuals evaluated with the EQ-5D-5L, 4 (20.00%) were treatment responders. A comparison of baseline characteristics between the EQ-5D-5L responder and non-responder groups revealed that the responders had significantly higher baseline KCPAT scores and significantly lower baseline EQ-5D-5L scores. No differences were found in the other variables (Table S5). Univariate logistic regression analysis of all covariates between these groups did not yield statistically significant results (Table S6).

No binary analysis was performed for the PainDETECT or KCPAT because of the absence of MCID values.

Univariate linear regression analysis of continuous assessment indicators, such as changes in PainDETECT, KCPAT, and EQ-5D-5L scores, identified baseline PainDETECT and KCPAT scores, occupation, and education as influencing factors, although caution is advised due to the small overall sample size (Table S7, Figure S1).

### 3.3. Safety Evaluation

A retrospective analysis of medical records revealed no adverse effects in the patients with successful follow-up.

## 4. Discussion

### 4.1. Summary of Findings

This retrospective study, analyzing data from 163 patients to evaluate the real-world clinical effectiveness of a combined ECA and ECLI therapy for common musculoskeletal pain. The patient cohort was diverse, with pain locations including the knee, back, neck, and shoulder.

Overall, the findings suggest that this combined treatment may offer a beneficial therapeutic strategy for musculoskeletal pain. Among the 44 participants with follow-up data, significant improvements were observed—in general, pain intensity and quality of life. Symptom-specific indices for knee pain and low back pain also showed significant improvements. However, the changes in NDI and SPADI scores were not statistically significant. Notably, a majority of the participants (59.0%) were identified as responders across various symptom-specific scores. Analysis of this responder group revealed that they were more frequent users of dietary supplements and had significantly lower baseline quality of life scores compared to non-responders.

This is the first study to demonstrate the effectiveness of this integrated treatment, providing a pioneering approach in TKM. These findings not only validate the clinical response but also offer a foundation for further studies on holistic and personalized medical practices.

### 4.2. Responder of ECM

In the context of ECM, it is crucial to consider variability in patient responses, as in other forms of TKM, including herbal and acupuncture treatments. Although our study did not find significant differences in this system between responders and non-responders, this aspect merits further discussion. The responder group had a markedly poorer quality of life at the start of treatment, suggesting that those with an overall more compromised physical condition might exhibit a more positive response to therapy. Furthermore, the higher rate of dietary supplement intake in the responder group may imply that superior health-related habits can lead to improved treatment outcomes. However, this hypothesis requires further investigation. Additional research is required to determine whether these tendencies are characteristic of acupuncture therapy, in general, or specific to this treatment. In the realm of herbal medicine, research has shown that factors such as BMI can influence treatment response [41] suggesting a need for personalized approaches in this system. Similarly, acupuncture studies have indicated variability in patient responses [42], underscoring the importance of individualized treatment plans. This highlights the potential for further investigation of the factors influencing responsiveness to this system, which could lead to more tailored and effective treatment strategies for musculoskeletal pain.

### 4.3. Personalized Medicine and ECM

The increasing awareness of personalized or precision medicine among clinicians, pharmaceutical companies, and medical consumers has underscored the need for tailored treatment approaches. Precision medicine typically involves the use of diagnostic tools and treatments tailored to the genetic, biomarker, or psychosocial characteristics of the patients [43]. This concept of patient-specific approaches predates the introduction of modern precision medicine and has long been a common strategy in TKM.

Within this framework, a key principle of personalization is the concept of a constitution, which classifies individuals into distinct types based on their unique physiological characteristics [44]. ECM, a unique constitutional medicine system in TKM, is a representative form of this personalization. Its diagnostic approach involves classifying individuals into eight inherent constitutions [45,46], with pulse diagnosis via radial artery palpation as a cornerstone [47]. Although historically qualitative, modern research, utilizing advanced measurement devices, increasingly validates its scientific understanding, reliability, and objectivity, affirming it as a valid and evolving diagnostic tool [48,49]. Building upon this broad framework of traditional pulse diagnosis, the standardized ECM pulse diagnostic technique evaluates the depth, width, and strength of the radial artery pulse, allowing classification into one of the eight constitutional types. Dietary and lifestyle intervention are then tailored to the individual’s constitution [50], sharing many goals with precision medicine and offering valuable insights for personalized healthcare [45].

### 4.4. Value and Strengths of ECM

Improvements in diet and exercise are crucial for enhancing health-related quality of life. However, the effects are not uniform and vary according to an individual’s constitution type [50]. This implies that the benefits of lifestyle changes for health promotion differ according to individual characteristics. ECM, which includes both ECA and ECLI, focuses on personalized, constitution-tailored dietary and lifestyle modifications such as exercise and bathing methods. Unlike TKM, which lacks extensive research on standardized treatment methods related to lifestyle interventions and tends to focus on dietary guidelines based on Sasang constitutional types, drugs, or psychological state management, this approach offers a standardized and detailed method for lifestyle improvement. The findings of this study suggest that this approach may be an effective therapeutic strategy for chronic metabolic diseases and pain disorders.

ECA uses a fast-in, fast-out method with standardized ECA guide tube, making it minimally invasive [22]. This aspect is particularly relevant considering the global trend towards minimally invasive, nonsurgical treatments for musculoskeletal pain, highlighting the high utility of this method [51]. Additionally, the diagnostic process in this system is highly standardized, with clinicians consistently using pulse diagnosis to classify patients into the same constitutional groups. This standardization of both diagnosis and treatment enhances treatment efficiency, accuracy, and predictability across various clinical situations [21].

### 4.5. Strengths and Implications for Clinical Practice

This study aimed to evaluate the treatment response and safety of ECA and ECLI for musculoskeletal pain in clinical practice setting. One of the main strengths is the use of real-world data from primary ECM clinics which retrospectively examined medical records of patients with various types of musculoskeletal pain. This is beneficial for assessing the treatment effectiveness and patient responses in real clinical settings. Although previous studies have used this model for neck pain, lumbar disc herniation, and degenerative knee osteoarthritis [17,19,52] were limited by small sample sizes, this is the first study to observe more than 150 participants. In contrast to previous studies on this treatment model, which primarily used outcomes such as the visual analog scale or Pain Disability Index [18,19,52,53], this study employed wider range of symptom-specific indices, such as PainDETECT, KCPAT, EQ5D5L, SPADI, NDI, ODI, and WOMAC. Moreover, this study is the first to explore groups showing clinically significant improvement and those that do not, using the MCID of each index to identify the factors influencing treatment effectiveness. Previous research on ECLI has shown significant improvements in general, social, and mental health; emotional role functioning; and psychological scores in groups with hepatonia/cholecystonia compared to pulmotonia/colonotonia [54]. However, that study was conducted at a single institution and was limited to a subset of constitutions. Therefore, this study, using this intervention, is the first to present the effect of specific musculoskeletal pain and quality of life in the general patient population, irrespective of specific constitutions. The results confirm that the combined ECA and ECLI can improve pain and quality of life in patients with musculoskeletal pain and highlight the potential of minimally invasive acupuncture to positively affect pain.

The ECM therapeutic approach is distinct from conventional pain management standards, including those of European guidelines [12]. While conventional care diagnoses pain based on a specific disease or anatomical damage and provides general recommendations, ECM finds the cause of pain in a patient’s unique physiological characteristics and constitution. In ECM, lifestyle interventions are not general but highly specific, providing tailored guidelines for diet, exercise, and bathing methods for each constitution. Furthermore, ECM systematically integrates acupuncture and constitution-based lifestyle interventions under a single diagnostic standard, a feature that distinguishes it from other multidisciplinary approaches. This unified diagnostic system, where all treatment elements are linked and interact organically, is the most unique feature. However, it is important to note that ECLI recommendations are designed within a specific cultural context, and acupuncture has a high acceptance in Korea due to national health insurance coverage. This highlights the need for further research to evaluate its effectiveness across diverse populations and healthcare systems.

Additionally, as there were no significant differences between the responders and non-responders, this approach can be applied to a diverse range of patients in clinical settings without being confined to any specific subgroup. Nevertheless, further research into the characteristics of the responder groups is necessary.

### 4.6. Limitation and Suggestion for Further Study

This study had several limitations. First, as a retrospective review of real-world data, a formal a priori power calculation was not performed. The study was exploratory, aiming to investigate clinical effectiveness based on existing medical records. We acknowledge this limitation and highlight the small sample size as a reason for caution when interpreting the results and as a justification for future large-scale prospective studies. Second, the retrospective chart review design cannot exclude the possibility of selection and information bias; therefore, caution should be exercised when interpreting the results of this study, especially regarding clinical effectiveness. The limited sample size also posed a challenge for statistical adjustment. Third, a retrospective analysis of real-world data may not have captured all the variables that could influence treatment outcomes. These findings provide foundational data and justification for future large-scale, prospective controlled studies, particularly for determining effect size, appropriate treatment duration, and outcome variable. Prospective studies are required to identify additional factors affecting treatment response. Our research team plans to conduct prospective registry studies based on these results. Fourth, this study followed the routine clinical practice protocols of ECM clinics by applying a combined intervention of acupuncture and lifestyle intervention, exploring the primary factors contributing to the observed effects challenging. Future studies should quantitatively evaluate dosage and adherence to this treatment and to lifestyle interventions. The development of assessment tools for lifestyle habits and dietary intake is a prerequisite for such studies. Fifth, the wide age range of participants may have introduced heterogeneity in treatment response. However, this reflects real-world clinical practice, where patients of various ages seek care for similar complaints, which may enhance the generalizability of the findings. Finally, its findings may have limited generalizability beyond East Asia, particularly to Western cultures. This is primarily due to the established public understanding and clinical integration of acupuncture and constitutional medicine in East Asian healthcare. Additionally, lifestyle management strategies like ECLI are culturally specific, making direct application elsewhere challenging. These contextual differences can significantly influence treatment outcomes. Therefore, caution is necessary when extrapolating results to different cultural or healthcare systems. Future research should explore the applicability of this treatment in diverse cultural settings.

## 5. Conclusions

To the best of our knowledge, this is the first study to report the clinical response and safety of individualized and minimally invasive treatment (ECA and ECLI) for musculoskeletal pain using real-world data. In this study, we suggest that these treatments can significantly improve certain metrics of musculoskeletal pain, indicating the potential for the clinical application of personalized constitution-based treatment through ECM. However, the limitations of this study, including its small sample size and retrospective design without a control group, necessitate further multi-institutional prospective studies and the development of tools to measure the dosage and adherence to this treatment.

## Figures and Tables

**Table 1:** Baseline characteristics of patients (n = 163).

Variables	Frequency (%)/Mean ± SD
By region (%)	
-Neck pain	62 (38.0%)
-Shoulder pain	26 (16.0%)
-Low back pain	49 (30.1%)
-Knee pain	26 (16.0%)
Sex	
-Female	118 (72.4%)
-Male	45 (27.6%)
Age	49.9 ± 14.3
-20s	11 (6.7%)
-30s	32 (19.6%)
-40s	30 (18.4%)
-50s	46 (28.2%)
-60s	29 (17.8%)
-70<	15 (9.2%)
Constitution diagnostic	
-Pulmotonia	10 (6.1%)
-Colonotonia	29 (17.8%)
-Hepatonia	34 (20.9%)
-Cholecystonia	24 (14.7%)
-Pancreotonia	36 (22.1%)
-Gastrotonia	1 (0.6%)
-Renotonia	8 (4.9%)
-Vesicotonia	10 (6.1%)
-Unknown	11 (6.7%)
Education	
-Elementary school	3 (1.8%)
-High school	28 (17.2%)
-Middle school	1 (0.6%)
-Bachelor’s degree/college	97 (59.5%)
-Master’s degree or higher	30 (18.4%)
-Unknown	4 (2.5%)
Marriage	
-No	40 (24.5%)
-Yes	118 (72.4%)
-Unknown	4 (2.5%)
-Others	1 (0.6%)
Job	
-Homemaker	42 (25.8%)
-Military personnel	2 (1.2%)
-Unemployed	12 (7.4%)
-Office worker	32 (19.6%)
-Service industry worker	11 (6.7%)
-Salesperson	1 (0.6%)
-Specialized occupation	32 (19.6%)
-Student	5 (3.1%)
-Unskilled laborer	3 (1.8%)
-Unknown	4 (2.5%)
-Others	19 (11.7%)
Alcohol consumption status	
-None	20 (12.3%)
-Yes	139 (85.3%)
-Unknown	4 (2.5%)
Total smoke exposure	
-Less than 5 packs	7 (4.3%)
-5 packs or more	37 (22.7%)
-Non-smoker	113 (69.3%)
-Unknown	4 (2.5%)
-Others (ex. e-cigarettes)	2 (1.2%)
Supplementary diet	
-No	75 (46.0%)
-Yes	88 (54.0%)
Baseline PainDETECT score	9.2 ± 5.4
Baseline KCPAT * score	11.7 ± 5.8
Baseline EQ-5D-5L score	0.7 ± 0.1

Categorical data are presented as frequency and ratio. Continuous data are presented as mean and standard deviation. * Only the somatic pain item scores from the KCPAT were used. EQ-5D-5L = EuroQol-5 dimension-5L; KCPAT = Korean Cancer Pain Assessment Tool; SD = Standard Deviation.

**Table 2:** Clinical response to ECM treatment on PainDETECT, KCPAT *, EQ5D-5L, each symptom-specific index.

Variable	Mean Differences (SD)	SMD	Pre (Mean (SD))	Post (Mean (SD))	*p*-Value **
PainDETECT (n = 44)	−0.39 (0.03)	−0.07	9.32 (4.76)	8.93 (5.06)	0.628
Neck pain (n = 10)	0.50 (6.70)	0.07	12.20 (3.43)	12.70 (3.43)	0.798
Shoulder pain (n = 7)	0.57 (4.20)	0.14	7.86 (4.88)	8.43 (3.26)	1.000
Low back pain (n = 19)	−1.89 (5.30)	−0.36	8.79 (3.43)	6.89 (5.03)	0.060
Knee pain (n = 8)	1.25 (3.62)	0.35	8.25 (4.80)	9.50 (5.98)	0.438
KCPAT * (n = 44)	−2.00 (5.69)	−0.35	11.77 (4.77)	9.77 (5.32)	0.024 ^†^
Neck pain (n = 10)	−0.80 (4.92)	−0.16	14.30 (4.47)	13.50 (4.70)	0.634
Shoulder pain (n = 7)	0.57 (7.98)	0.07	10.71 (6.18)	11.29 (6.16)	0.916
Low back pain (n = 19)	−3.32 (6.06)	−0.55	11.68 (4.70)	8.37 (4.69)	0.045 ^‡^
Knee pain (n = 8)	−2.63 (2.20)	−1.19	9.75 (3.11)	7.13 (4.55)	0.034 ^‡^
EQ-5D-5L (n = 20)	0.07 (0.11)	0.63	0.74 (0.12)	0.80 (0.07)	0.012 ^‡^
NDI (n = 10)	−0.89 (12.32)	−0.07	28.60 (16.54)	27.71 (8.58)	1.000
SPADI (n = 7)	−1.10 (8.95)	−0.12	45.93 (22.81)	44.84 (16.97)	0.588
ODI (n = 19)	−32.81 (57.54)	−0.57	155.38 (51.69)	122.57 (79.40)	0.019 ^‡^
WOMAC (n = 8)	−14.63 (15.55)	−0.94	46.13 (18.08)	31.50 (20.24)	0.042 ^‡^

* Only the somatic pain item scores from the KCPAT were used. ** For sample sizes < 30, the non-parametric Wilcoxon Signed-Rank Test was performed, and for sample sizes of ≥30 that satisfied the normality test, a paired *t*-test was performed. ^†^ Statistically significant decrease from baseline (*p* using paired *t*-test). ^‡^ Statistically significant decrease from baseline (*p* using Wilcoxon Signed-Rank Test). EQ-5D-5L = EuroQol-5 dimension-5L; KCPAT = Korean Cancer Pain Assessment Tool; NDI = Neck Disability Index; ODI = Oswestry Disability Index; SD = Standard Deviation; SMD = Standardized Mean Difference; SPADI = Shoulder Pain and disability Index; WOMAC = Western Ontario and McMaster Universities Osteoarthritis Index.

## Data Availability

The datasets used and/or analyzed during the current study are available from the corresponding author on reasonable request.

## Supplementary Materials

The following supporting information can be downloaded at https://www.mdpi.com/article/10.3390/medicina61091564/s1, Figure S1: Analysis of covariates influencing the changes in PainDETECT and partial KCPAT scores; Table S1: Baseline characteristics of patients by pain region (n = 163); Table S2: Comparative baseline characteristics of successful and failed follow-up groups; Table S3: Baseline characteristics according to follow-up success; Table S4: Clinical response to ECM treatment by Age Group; PainDETECT and KCPAT Scores; Table S5: Baseline demographic and clinical characteristics of responders versus non-responders; Table S6: Logistic regression predicting the odds of response after treatment; Table S7: Analysis of covariates influencing the changes in PainDETECT and partial KCPAT scores; File S1: The ECLI plan.

## Abbreviations

The following abbreviations are used in this manuscript:

ECM	Eight Constitution Medicine
ECA	Eight Constitution Acupuncture
ECLI	Eight Constitution Lifestyle Intervention
KCPAT	Korean Cancer Pain Assessment Tool
KMD	Korean Medicine Doctor
WOMAC	Western Ontario and McMaster Universities Osteoarthritis Index
ODI	Oswestry Disability Index
NDI	Neck Disability Index
SPADI	Shoulder Pain and Disability Index
TKM	Traditional Korean Medicine
LBP	Low Back Pain
EATM	East Asian Traditional Medicine
PRO	Patient-Reported Outcomes
MCID	Minimal Clinically Important Difference
IRB	Institutional Review Board
