# Supplementary material for: Pain Treatment in Primary Care Through Eight Constitution Medicine: A Retrospective Real-World Study from South Korea
Source: Medicina (Kaunas). 2025 Aug 30;61(9):1564. doi: 10.3390/medicina61091564 (PMC12472020; doi:10.3390/medicina61091564)
Supplement: Supplementary file 1 [file medicina-61-01564-s001.zip › supplementary figures and tables_0805_track change (-).pdf]

Table S1. Baseline characteristics of patients by pain region (n = 163)

By region	Neck pain (N=62)	Shoulder pain (N=26)	Low back pain (N=49)	Knee pain (N=26)
**Follow-up**				
- Fail	52 (83.9%)	19 (73.1%)	30 (61.2%)	18 (69.2%)
- Success	10 (16.1%)	7 (26.9%)	19 (38.8%)	8 (30.8%)
**Sex**				
- Female	42 (67.7%)	22 (84.6%)	34 (69.4%)	20 (76.9%)
- Male	20 (32.3%)	4 (15.4%)	15 (30.6%)	6 (23.1%)
**Age**	45.4 ± 13.0	53.8 ± 10.1	50.7 ± 16.3	55.0 ± 14.1
**Constitution diagnostic**				
- Pulmotonia	6 (9.7%)	1 (3.8%)	1 (2.0%)	2 (7.7%)
- Colonotonia	12 (19.4%)	7 (26.9%)	9 (18.4%)	1 (3.8%)
- Hepatonia	8 (12.9%)	6 (23.1%)	11 (22.4%)	9 (34.6%)
- Cholecystonia	8 (12.9%)	2 (7.7%)	11 (22.4%)	3 (11.5%)
- Pancreotonia	14 (22.6%)	5 (19.2%)	9 (18.4%)	8 (30.8%)
- Gastrotonia	0 (0.0%)	1 (3.8%)	0 (0.0%)	0 (0.0%)
- Renotonia	4 (6.5%)	0 (0.0%)	4 (8.2%)	0 (0.0%)
- Vesicotonia	4 (6.5%)	2 (7.7%)	3 (6.1%)	1 (3.8%)
- Unknown	6 (9.7%)	2 (7.7%)	1 (2.0%)	2 (7.7%)
**Education**				
- Elementary school	0 (0.0%)	2 (7.7%)	1 (2.0%)	0 (0.0%)
- High school	8 (12.9%)	5 (19.2%)	9 (18.4%)	6 (23.1%)
- Middle school	0 (0.0%)	0 (0.0%)	1 (2.0%)	0 (0.0%)
- Bachelor's degree / college	38 (61.3%)	14 (53.8%)	28 (57.1%)	17 (65.4%)
- Master’s degree or higher	15 (24.2%)	3 (11.5%)	10 (20.4%)	2 (7.7%)
- Unknown	1 (1.6%)	2 (7.7%)	0 (0.0%)	1 (3.8%)
**Marriage**				
- No	19 (30.6%)	1 (3.8%)	14 (28.6%)	6 (23.1%)
- Yes	42 (67.7%)	23 (88.5%)	34 (69.4%)	19 (73.1%)
- Unknown	1 (1.6%)	2 (7.7%)	0 (0.0%)	1 (3.8%)
- Others	0 (0.0%)	0 (0.0%)	1 (2.0%)	0 (0.0%)
**Job**				
- Homemaker	10 (16.1%)	8 (30.8%)	14 (28.6%)	10 (38.5%)
- Military personnel	1 (1.6%)	0 (0.0%)	0 (0.0%)	1 (3.8%)
- Unemployed	3 (4.8%)	2 (7.7%)	4 (8.2%)	3 (11.5%)
- Office worker	16 (25.8%)	5 (19.2%)	8 (16.3%)	3 (11.5%)
- Service industry worker	3 (4.8%)	2 (7.7%)	5 (10.2%)	1 (3.8%)
- Salesperson	1 (1.6%)	0 (0.0%)	0 (0.0%)	0 (0.0%)

By region	Neck pain (N=62)	Shoulder pain (N=26)	Low back pain (N=49)	Knee pain (N=26)
- Specialized occupation	17 (27.4%)	5 (19.2%)	6 (12.2%)	4 (15.4%)
- Student	1 (1.6%)	0 (0.0%)	4 (8.2%)	0 (0.0%)
- Unskilled laborer	1 (1.6%)	0 (0.0%)	2 (4.1%)	0 (0.0%)
- Unknown	1 (1.6%)	2 (7.7%)	0 (0.0%)	1 (3.8%)
- Others	8 (12.9%)	2 (7.7%)	6 (12.2%)	3 (11.5%)
**Alcohol consumption status**				
- None	4 (6.5%)	5 (19.2%)	6 (12.2%)	5 (19.2%)
- Yes	57 (91.9%)	19 (73.1%)	43 (87.8%)	20 (76.9%)
- Unknown	1 (1.6%)	2 (7.7%)	0 (0.0%)	1 (3.8%)
**Total smoke exposure**				
- Others (ex. e-cigarettes)"	2 (3.2%)	0 (0.0%)	0 (0.0%)	0 (0.0%)
- Less than 5 packs	2 (3.2%)	0 (0.0%)	4 (8.2%)	1 (3.8%)
- 5 packs or more	16 (25.8%)	4 (15.4%)	12 (24.5%)	5 (19.2%)
- Non-smoker	41 (66.1%)	20 (76.9%)	33 (67.3%)	19 (73.1%)
- Unknown	1 (1.6%)	2 (7.7%)	0 (0.0%)	1 (3.8%)
**Supplementary diet**				
- No	29 (46.8%)	11 (42.3%)	21 (42.9%)	14 (53.8%)
- Yes	33 (53.2%)	15 (57.7%)	28 (57.1%)	12 (46.2%)
**Baseline PainDETECT score**	9.9 ± 6.0	9.3 ± 3.9	8.2 ± 4.6	9.3 ± 6.5
**Baseline KCPAT** score**	12.7 ± 6.1	11.2 ± 6.1	11.3 ± 5.4	10.2 ± 5.2
**Baseline EQ-5D-5L score**	0.8 ± 0.1	0.8 ± 0.1	0.7 ± 0.1	0.7 ± 0.1
**symptom-specific index ***	27.7 ± 12.4	52.4 ± 24.0	135.4 ± 54.6	40.0 ± 18.2

* A symptom-specific index refers to one of the following: Vernon-Mior Neck Disability Index (NDI), Oswestry Disability Index (ODI), Western Ontario and McMaster Universities Osteoarthritis Index (WOMAC), or Shoulder Pain and Disability Index (SPADI).

** Only the somatic pain item scores from the KCPAT were used.

Continuous Data are expressed as mean±standard deviation. Categorical data are expressed as frequency and ratio

EQ-5D-5L = EuroQol-5 dimension-5L; KCPAT = Korean Cancer Pain Assessment Tool

Table S2. Comparative baseline characteristics of successful and failed follow-up groups.

Follow-up	Fail (n=119)	Success (n=44)
By region (%)		
- Neck pain	52 (43.7)	10 (22.7)
- Shoulder pain	19 (16.0)	7 (15.9)
- Low back pain	30 (25.2)	19 (43.2)
- Knee pain	18 (15.1)	8 (18.2)
Age (mean (SD))	48.4 ± 15.0	53.9 ± 11.4
- 20s (%)	11 (100.0)	0 (0.0)
- 30s (%)	27 (84.4)	5 (15.6)
- 40s (%)	22 (73.3)	8 (26.7)
- 50s (%)	28 (60.9)	18 (39.1)
- 60s (%)	20 (69.0)	9 (31.0)
- 70< (%)	11 (73.3)	4 (26.7)
Sex = Male (%)	34 (28.6)	11 (25.0)
Constitution diagnostic (%)		
- Pulmotonia	8 (6.7)	2 (4.5)
- Colonotonia	23 (19.3)	6 (13.6)
- Hepatonia	24 (20.2)	10 (22.7)
- Cholecystonia	15 (12.6)	9 (20.5)
- Pancreotonia	27 (22.7)	9 (20.5)
- Gastrotonia	1 (0.8)	0 (0.0)
- Renotonia	5 (4.2)	3 (6.8)
- Vesicotonia	7 (5.9)	3 (6.8)
- Unknown	9 (7.6)	2 (4.5)
Education (%)		
- Elementary school	3 (2.5)	0 (0.0)
- High school	19 (16.0)	9 (20.5)
- Middle school	0 (0.0)	1 (2.3)
- Bachelor's degree	71 (59.7)	26 (59.1)
- Master's degree or higher	22 (18.5)	8 (18.2)
- Unknown	4 (3.4)	0 (0.0)
Marriage (%)		
- No	32 (26.9)	8 (18.2)
- Yes	83 (69.7)	35 (79.5)
- Unknown	4 (3.4)	0 (0.0)
- Others	0 (0.0)	1 (2.3)
Job (%)		
- Homemaker	24 (20.2)	18 (40.9)
- Military personnel	2 (1.7)	0 (0.0)
- Unemployed	9 (7.6)	3 (6.8)
- Office worker	24 (20.2)	8 (18.2)

- Service industry worker	7 (5.9)	4 (9.1)
- Salesperson	1 (0.8)	0 (0.0)
- Specialized occupation	28 (23.5)	4 (9.1)
- Student	5 (4.2)	0 (0.0)
- Unskilled laborer	1 (0.8)	2 (4.5)
- Unknown	4 (3.4)	0 (0.0)
- Others	14 (11.8)	5 (11.4)
Alcohol consumption status (%)		
- None	12 (10.1)	8 (18.2)
- Yes	103 (86.6)	36 (81.8)
- Unknown	4 (3.4)	0 (0.0)
Total smoke exposure (%)		
- Less than 5 packs	7 (5.9)	0 (0.0)
- 5 packs or more	26 (21.8)	11 (25.0)
- Non-smoker	80 (67.2)	33 (75.0)
- Unknown	4 (3.4)	0 (0.0)
- Others (ex. e-cigarettes)"	2 (1.7)	0 (0.0)
Suppelmentary diet = Yes (%)	60 (50.4)	28 (63.6)
Baseline PainDETECT score (mean (SD))	9.0 [5.0;13.0]	9.0 [6.0;12.0]
Baseline KCPAT **score (mean (SD))	11.0 [6.0;16.0]	11.5 [8.5;14.0]
Baseline EQ-5D-5L score (mean (SD))	0.8 [0.7; 0.8]	0.8 [0.7; 0.8]
Baseline WOMAC score (mean (SD))	37.22 (18.00)	46.12 (18.08)
Baseline ODI score (mean (SD))	122.70 (53.33)	155.38 (51.69)
Baseline NDI score (mean (SD))	27.51 (11.65)	28.60 (16.54)
Baseline SPADI score (mean (SD))	54.84 (24.51)	45.93 (22.81)

Continuous data are expressed as mean (standard deviation); if the data do not satisfy a normal distribution, they are expressed as median (Interquartile Range). Categorical data are expressed as frequencies and ratios.

* For continuous variables, the Shapiro-Wilk normality test was conducted. If the distribution was normal, an independent t-test was performed; otherwise, the Mann-Whitney U test was performed.

**Only the somatic pain item scores from the KCPAT were used. †Statistically significantly decreased from baseline (p using independent t-test). EQ-5D-5L = EuroQol-5 dimension-5L; KCPAT = Korean Cancer Pain Assessment Tool ; NDI = vernon-mior neck disability index; ODI = Oswestry Disability Index; SD=Standard Deviation;, SPADI = Shoulder Pain and disability Index; WOMAC =Western Ontario and McMaster Universities Osteoarthritis Index.

Table S3. Baseline characteristics according to follow-up success

Follow-up	Success (N=44)					Fail (N=119)				
By Region	Neck Pain	Shoulder Pain	Low Back Pain	Knee Pain	p†	Neck Pain	Shoulder Pain	Low Back Pain	Knee Pain	p†
	(N=10)	(N=7)	(N=19)	(N=8)		(N=52)	(N=19)	(N=30)	(N=18)	
**Sex**					0.709					0.244
- Female	7 (70.0%)	6 (85.7%)	15 (78.9%)	5 (62.5%)		35 (67.3%)	16 (84.2%)	19 (63.3%)	15 (83.3%)	
- Male	3 (30.0%)	1 (14.3%)	4 (21.1%)	3 (37.5%)		17 (32.7%)	3 (15.8%)	11 (36.7%)	3 (16.7%)	
**Age**	53.0 ± 10.3	55.3 ± 6.5	52.2 ± 13.5	58.0 ± 11.1	0.664	43.9 ± 13.0	53.3 ± 11.2	49.8 ± 18.0	53.7 ± 15.4	0.025‡
**- 20s**	-	-	-	-	-	5 (45.5%)	0 (0.0%)	5 (45.5%)	1 (9.1%)	
**- 30s**	0 (0.0%)	0 (0.0%)	4 (80.0%)	1 (20.0%)		17 (63.0%)	2 (7.4%)	5 (18.5%)	3 (11.1%)	
**- 40s**	4 (50.0%)	1 (12.5%)	3 (37.5%)	0 (0.0%)		13 (59.1%)	5 (22.7%)	3 (13.6%)	1 (4.5%)	
**- 50s**	4 (22.2%)	5 (27.8%)	6 (33.3%)	3 (16.7%)		9 (32.1%)	9 (32.1%)	5 (17.9%)	5 (17.9%)	
**- 60s**	1 (11.1%)	1 (11.1%)	3 (33.3%)	4 (44.4%)		5 (25.0%)	1 (5.0%)	9 (45.0%)	5 (25.0%)	
**- 70<**	1 (25.0%)	0 (0.0%)	3 (75.0%)	0 (0.0%)		3 (27.3%)	2 (18.2%)	3 (27.3%)	3 (27.3%)	
**Constitution diagnostic**					1					0.48
- Pulmotonia	1 (10.0%)	0 (0.0%)	0 (0.0%)	1 (12.5%)		5 (9.6%)	1 (5.3%)	1 (3.3%)	1 (5.6%)	
- Colonotonia	2 (20.0%)	2 (28.6%)	2 (10.5%)	0 (0.0%)		10 (19.2%)	5 (26.3%)	7 (23.3%)	1 (5.6%)	
- Hepatonia	3 (30.0%)	1 (14.3%)	4 (21.1%)	2 (25.0%)		5 (9.6%)	5 (26.3%)	7 (23.3%)	7 (38.9%)	
- Cholecystonia	2 (20.0%)	1 (14.3%)	5 (26.3%)	1 (12.5%)		6 (11.5%)	1 (5.3%)	6 (20.0%)	2 (11.1%)	
- Pancreotonia	0 (0.0%)	2 (28.6%)	4 (21.1%)	3 (37.5%)		14 (26.9%)	3 (15.8%)	5 (16.7%)	5 (27.8%)	
- Gastrotonia	0 (0.0%)	0 (0.0%)	0 (0.0%)	0 (0.0%)		0 (0.0%)	1 (5.3%)	0 (0.0%)	0 (0.0%)	
- Renotonia	0 (0.0%)	0 (0.0%)	3 (15.8%)	0 (0.0%)		4 (7.7%)	0 (0.0%)	1 (3.3%)	0 (0.0%)	
- Vesicotonia	1 (10.0%)	1 (14.3%)	1 (5.3%)	0 (0.0%)		3 (5.8%)	1 (5.3%)	2 (6.7%)	1 (5.6%)	
- Unknown	1 (10.0%)	0 (0.0%)	0 (0.0%)	1 (12.5%)		5 (9.6%)	2 (10.5%)	1 (3.3%)	1 (5.6%)	
**Education**					1					1
- Elementary school	0 (0.0%)	0 (0.0%)	0 (0.0%)	0 (0.0%)		0 (0.0%)	2 (10.5%)	1 (3.3%)	0 (0.0%)	
- High school	3 (30.0%)	1 (14.3%)	3 (15.8%)	2 (25.0%)		5 (9.6%)	4 (21.1%)	6 (20.0%)	4 (22.2%)	
- Middle school	0 (0.0%)	0 (0.0%)	1 (5.3%)	0 (0.0%)		0 (0.0%)	0 (0.0%)	0 (0.0%)	0 (0.0%)	
- Bachelor's degree /college	5 (50.0%)	6 (85.7%)	9 (47.4%)	6 (75.0%)		33 (63.5%)	8 (42.1%)	19 (63.3%)	11 (61.1%)	
- Master’s degree or higher	2 (20.0%)	0 (0.0%)	6 (31.6%)	0 (0.0%)		13 (25.0%)	3 (15.8%)	4 (13.3%)	2 (11.1%)	
- Unknown	0 (0.0%)	0 (0.0%)	0 (0.0%)	0 (0.0%)		1 (1.9%)	2 (10.5%)	0 (0.0%)	1 (5.6%)	
**Marriage**					1					1
- No	2 (20.0%)	1 (14.3%)	4 (21.1%)	1 (12.5%)		17 (32.7%)	0 (0.0%)	10 (33.3%)	5 (27.8%)	

Follow-up	Success (N=44)					Fail (N=119)				
By Region	Neck Pain	Shoulder Pain	Low Back Pain	Knee Pain	p†	Neck Pain	Shoulder Pain	Low Back Pain	Knee Pain	p†
	**(N=10)**	**(N=7)**	**(N=19)**	**(N=8)**		**(N=52)**	**(N=19)**	**(N=30)**	**(N=18)**	
- Yes	8 (80.0%)	6 (85.7%)	14 (73.7%)	7 (87.5%)		34 (65.4%)	17 (89.5%)	20 (66.7%)	12 (66.7%)	
- Unknown	0 (0.0%)	0 (0.0%)	0 (0.0%)	0 (0.0%)		1 (1.9%)	2 (10.5%)	0 (0.0%)	1 (5.6%)	
- Others	0 (0.0%)	0 (0.0%)	1 (5.3%)	0 (0.0%)		0 (0.0%)	0 (0.0%)	0 (0.0%)	0 (0.0%)	
**Job**					1					0.406
- Homemaker	3 (30.0%)	3 (42.9%)	7 (36.8%)	5 (62.5%)		7 (13.5%)	5 (26.3%)	7 (23.3%)	5 (27.8%)	
- Military personnel	0 (0.0%)	0 (0.0%)	0 (0.0%)	0 (0.0%)		1 (1.9%)	0 (0.0%)	0 (0.0%)	1 (5.6%)	
- Unemployed	1 (10.0%)	1 (14.3%)	1 (5.3%)	0 (0.0%)		2 (3.8%)	1 (5.3%)	3 (10.0%)	3 (16.7%)	
- Office worker	4 (40.0%)	2 (28.6%)	1 (5.3%)	1 (12.5%)		12 (23.1%)	3 (15.8%)	7 (23.3%)	2 (11.1%)	
- Service industry worker	1 (10.0%)	0 (0.0%)	3 (15.8%)	0 (0.0%)		2 (3.8%)	2 (10.5%)	2 (6.7%)	1 (5.6%)	
- Salesperson	0 (0.0%)	0 (0.0%)	0 (0.0%)	0 (0.0%)		1 (1.9%)	0 (0.0%)	0 (0.0%)	0 (0.0%)	
- Specialized occupation	0 (0.0%)	0 (0.0%)	2 (10.5%)	2 (25.0%)		17 (32.7%)	5 (26.3%)	4 (13.3%)	2 (11.1%)	
- Student	0 (0.0%)	0 (0.0%)	0 (0.0%)	0 (0.0%)		1 (1.9%)	0 (0.0%)	4 (13.3%)	0 (0.0%)	
- Unskilled laborer	0 (0.0%)	0 (0.0%)	2 (10.5%)	0 (0.0%)		1 (1.9%)	0 (0.0%)	0 (0.0%)	0 (0.0%)	
- Unknown	0 (0.0%)	0 (0.0%)	0 (0.0%)	0 (0.0%)		1 (1.9%)	2 (10.5%)	0 (0.0%)	1 (5.6%)	
- Others	1 (10.0%)	1 (14.3%)	3 (15.8%)	0 (0.0%)		7 (13.5%)	1 (5.3%)	3 (10.0%)	3 (16.7%)	
**Alcohol consumption status**					1					0.094
- None	2 (20.0%)	1 (14.3%)	3 (15.8%)	2 (25.0%)		2 (3.8%)	4 (21.1%)	3 (10.0%)	3 (16.7%)	
- Yes	8 (80.0%)	6 (85.7%)	16 (84.2%)	6 (75.0%)		49 (94.2%)	13 (68.4%)	27 (90.0%)	14 (77.8%)	
- Unknown	0 (0.0%)	0 (0.0%)	0 (0.0%)	0 (0.0%)		1 (1.9%)	2 (10.5%)	0 (0.0%)	1 (5.6%)	
**Total smoke exposure**					1					0.189
- Others (ex. e-cigarettes)"	0 (0.0%)	0 (0.0%)	0 (0.0%)	0 (0.0%)		2 (3.8%)	0 (0.0%)	0 (0.0%)	0 (0.0%)	
- Less than 5 packs	0 (0.0%)	0 (0.0%)	0 (0.0%)	0 (0.0%)		2 (3.8%)	0 (0.0%)	4 (13.3%)	1 (5.6%)	
- 5 packs or more	4 (40.0%)	3 (42.9%)	3 (15.8%)	1 (12.5%)		12 (23.1%)	1 (5.3%)	9 (30.0%)	4 (22.2%)	
- Non-smoker	6 (60.0%)	4 (57.1%)	16 (84.2%)	7 (87.5%)		35 (67.3%)	16 (84.2%)	17 (56.7%)	12 (66.7%)	
- Unknown	0 (0.0%)	0 (0.0%)	0 (0.0%)	0 (0.0%)		1 (1.9%)	2 (10.5%)	0 (0.0%)	1 (5.6%)	
**Supplementary diet**					0.533					0.492
- No	4 (40.0%)	4 (57.1%)	5 (26.3%)	3 (37.5%)		25 (48.1%)	7 (36.8%)	16 (53.3%)	11 (61.1%)	
- Yes	6 (60.0%)	3 (42.9%)	14 (73.7%)	5 (62.5%)		27 (51.9%)	12 (63.2%)	14 (46.7%)	7 (38.9%)	
**Baseline PainDETECT score**	12.2 ± 6.2	7.9 ± 4.9	8.8 ± 3.4	8.2 ± 4.8	0.175	9.0 [4.5;14.0]	9.0 [9.0;12.5]	7.0 [4.0;11.0]	7.0 [5.0;15.0]	0.421
**Baseline KCPAT** score**	12.5 [12.0;16.0]	11.0 [5.0;15.5]	11.0 [7.5;14.0]	10.0 [8.0;12.5]	0.208	10.5 [8.0;17.0]	12.0 [6.0;17.0]	11.0 [6.0;15.0]	11.0 [5.0;14.0]	0.672

Follow-up	Success (N=44)					Fail (N=119)				
By Region	Neck Pain	Shoulder Pain	Low Back Pain	Knee Pain	p†	Neck Pain	Shoulder Pain	Low Back Pain	Knee Pain	p†
	**(N=10)**	**(N=7)**	**(N=19)**	**(N=8)**		**(N=52)**	**(N=19)**	**(N=30)**	**(N=18)**	
**Baseline EQ-5D-5L score**	0.8 [0.7; 0.8]	0.8 [0.8; 0.8]	0.8 [0.7; 0.8]	0.8 [0.7; 0.8]	0.531	0.8 [0.7; 0.9]	0.8 [0.7; 0.8]	0.8 [0.7; 0.8]	0.7 [0.7; 0.8]	0.287
**Symptom-specific index at baseline ***	25.2 [17.8;46.0]	56.2 [34.2;60.0]	160.0 [120.0;180.0]	47.5 [31.0;63.5]	0	27.3 [18.0;34.8]	50.0 [38.5;75.0]	105.0 [90.0;160.0]	37.5 [24.0;50.0]	0

* A symptom-specific index refers to one of the following: Vernon-Mior Neck Disability Index (NDI), Oswestry Disability Index (ODI), Western Ontario and McMaster Universities Osteoarthritis Index (WOMAC), or Shoulder Pain and Disability Index (SPADI).

** Only the somatic pain item scores from the KCPAT were used.

Continuous data are expressed as mean (standard deviation). If the data do not satisfy a normal distribution, they are expressed as medians [Interquartile Range]. Categorical data are expressed as frequencies and ratios.

† For continuous variables, the Shapiro-Wilks normality test was performed. If normally distributed, ANOVA was performed; if not normally distributed, the Kruskal-Wallis Rank Sum Test was performed.

‡ Statistically significantly decreased from baseline (p using ANOVA)

KCPAT = Korean Cancer Pain Assessment Tool , EQ-5D-5L = EuroQol-5 dimension-5L

Table S4. Clinical response to ECM treatment by Age Group; PainDETECT and KCPAT* Scores

variable	Mean differences (SD)	SMD	Pre (mean (SD))	Post (mean (SD))	p-value**
PainDETECT (n=44)	-0.39 (0.03)	-0.07	9.32 (4.76)	8.93 (5.06)	0.628
30s (n=5)	-2.80 (4.32)	-0.65	7.60 (4.28)	4.80 (3.83)	0.221
40s (n=8)	-2.50 (6.46)	-0.36	12.25 (6.88)	9.75 (4.62)	0.31
50s (n=18)	0.61 (5.05)	0.14	8.39 (4.53)	9.00 (4.49)	0.615
60s (n=9)	-0.11 (4.17)	-0.03	9.89 (3.55)	9.78 (6.00)	0.938
70< (n=4)	1.75 (6.95)	0.74	8.50 (2.38)	10.25 (7.27)	0.649
KCPAT*(n=44)	-2.00 (5.69)	-0.35	11.77 (4.77)	9.77 (5.32)	**0.024†**
30s (n=5)	-2.60 (2.41)	-0.39	9.40 (6.66)	6.80 (5.12)	0.073
40s (n=8)	-2.00 (6.61)	-0.42	15.62 (4.75)	13.62 (6.21)	0.421
50s (n=18)	-1.67 (6.32)	-0.37	11.33 (4.56)	9.67 (4.63)	0.279
60s (n=9)	-3.22 (4.63)	-0.85	10.56 (3.78)	7.33 (4.18)	0.07
70< (n=4)	0.00 (7.70)	0.0	11.75 (2.06)	11.75 (5.91)	1.0

*The total KCPAT score was used, not limited to somatic pain item scores. ** Paired t-tests were used for all comparisons, and normality assumptions were not tested. † Statistically significant decrease from baseline ($p < 0.05$ using paired t-test).

Table S5. Baseline demographic and clinical characteristics of responders versus non-responders.

	EQ-5D-5L score non-responder (n=16)	EQ-5D-5L score responder (n=4)	p†	Symptom-specific index * non-responder (n=18)	Symptom-specific index * responder (n=26)	p†
Age (median [IQR])	54.00 [40.75, 58.00]	51.00 [47.25, 53.00]	0.602	53.00 [50.00, 60.50]	53.50 [43.75, 61.75]	0.658
**Age (%)**	**-**	**-**	**0.603**	**-**	**-**	**0.109**
- 20s	0 (0.0%)	0 (0.0%)		0 (0.0%)	0 (0.0%)	
- 30s	3 (18.8%)	0 (0.0%)		0 (0.0%)	5 (19.2%)	
- 40s	4 (25.0%)	2 (50.0%)		4 (22.2%)	4 (15.4%)	
- 50s	5 (31.2%)	2 (50.0%)		9 (50.0%)	9 (34.6%)	
- 60s	3 (18.8%)	0 (0.0%)		2 (11.1%)	7 (26.9%)	
- 70<	1 (6.2%)	0 (0.0%)		3 (16.7%)	1 (3.8%)	
By region (%)			0.354			0.093
- Neck pain	2 (12.5)	2 (50.0)		6 (33.3)	4 (15.4)	
- Shoulder pain	1 (6.2)	0 (0.0)		5 (27.8)	2 (7.7)	
- Low back pain	8 (50.0)	2 (50.0)		5 (27.8)	14 (53.8)	
- Knee pain	5 (31.2)	0 (0.0)		2 (11.1)	6 (23.1)	
Constitution diagnostic (%)			0.818	18 (100.0)	26 (100.0)	0.902
- Pulmotonia	1 (6.2)	1 (25.0)		1 (5.6)	1 (3.8)	
- Colonotonia	1 (6.2)	1 (25.0)		3 (16.7)	6 (23.1)	
- Hepatonia	4 (25.0)	0 (0.0)		6 (33.3)	4 (15.4)	
- Cholecystonia	4 (25.0)	1 (25.0)		0 (0.0)	2 (7.7)	
- Pancreotonia	2 (12.5)	0 (0.0)		4 (22.2)	5 (19.2)	
- Gastrotonia	3 (18.8)	1 (25.0)		2 (11.1)	4 (15.4)	
- Renotonia	1 (6.2)	0 (0.0)		1 (5.6)	2 (7.7)	
- Unknown	0 (0.0)	0 (0.0)		0 (0.0)	0 (0.0)	
Sex = Male (%)	8 (50.0)	1 (25.0)	0.591	4 (22.2)	7 (26.9)	1
Education (%)			0.818			0.487
- Elementary school	1 (6.2)	0 (0.0)		0 (0.0)	1 (3.8)	
- High school	3 (18.8)	1 (25.0)		4 (22.2)	4 (15.4)	
- Middle school	10 (62.5)	2 (50.0)		12 (66.7)	14 (53.8)	
- Unknown	2 (12.5)	1 (25.0)		2 (11.1)	7 (26.9)	
Marriage (%)			0.538			0.823
- No	4 (25.0)	0 (0.0)		4 (22.2)	4 (15.4)	
- Yes	12 (75.0)	4 (100.0)		14 (77.8)	21 (80.8)	
- Others	0 (0.0)	0 (0.0)		0 (0.0)	1 (3.8)	
Job (%)			0.566			0.694
- Homemaker	3 (18.8)	3 (75.0)		7 (38.9)	11 (42.3)	
- Unemployed	0 (0.0)	0 (0.0)		1 (5.6)	2 (7.7)	
- Office worker	4 (25.0)	1 (25.0)		5 (27.8)	3 (11.5)	
- Service industry worker	1 (6.2)	0 (0.0)		1 (5.6)	3 (11.5)	
- Specialized Occupation	3 (18.8)	0 (0.0)		1 (5.6)	3 (11.5)	
- Unskilled laborer	2 (12.5)	0 (0.0)		0 (0.0)	2 (7.7)	
- Others	3 (18.8)	0 (0.0)		3 (16.7)	2 (7.7)	
Alcohol consumption status = Yes(%)	14 (87.5)	3 (75.0)	0.509	14 (77.8)	22 (84.6)	0.697
Total smoke exposure = No (%)	13 (81.2)	3 (75.0)	1.000	11 (61.1)	22 (84.6)	0.093
Supplementary diet = Yes (%)	10 (62.5)	4 (100.0)	0.267	7 (38.9)	21 (80.8)	0.01‡

Baseline PainDETECT score (median [IQR])	7.50 [6.00, 9.00]	9.50 [8.50, 12.50]	0.116	8.50 [6.00, 11.75]	9.00 [7.00, 12.00]	0.565
Baseline KCPAT **score (median [IQR])	9.00 [7.00, 11.25]	13.50 [11.75, 16.50]	0.028‡	12.00 [11.00, 14.00]	10.50 [7.00, 13.75]	0.236
Baseline EQ-5D-5L score (median [IQR])	0.77 [0.75, 0.82]	0.51 [0.49, 0.59]	0.047‡	0.81 [0.78, 0.85]	0.74 [0.65, 0.80]	0.018‡

* The term “symptom-specific index” refers to one of the following: NDI, ODI, WOMAC, or SPADI.

** Only the somatic pain item scores from the KCPAT were used.

† All continuous variables were tested using the Kruskal-Wallis test, and all categorical variables were tested using the Fisher’s exact test or chi-square test.

‡ Statistically significantly different from non-responder group.

EQ-5D-5L = EuroQol-5 dimension-5L; IQR=interquartile ragne; KCPAT = Korean Cancer Pain Assessment Tool ; NDI = vernon-mior neck disability index; ODI = Oswestry Disability Index; SPADI = Shoulder Pain and disability Index; WOMAC =Western Ontario and McMaster Universities Osteoarthritis Index

Continuous data are expressed as mean (standard deviation). If the data do not satisfy a normal distribution, they are expressed as median [Interquartile Range]. Categorical data are expressed as frequency and ratio.

Table S6. Logistic regression predicting the odds of response after treatment

	EQ-5D-5L responder versus non-responder				Symptom-specific index responder versus non-responder			
**Characteristic**	**N**	**OR***	**95% CI***	**p-value**	**N**	**OR***	**95% CI***	**p-value**
**Sex**	20	0.33	0.01, 3.27	0.4	44	1.29	0.32, 5.73	0.7
**Age**	20	0.99	0.88, 1.10	0.8	44	0.98	0.92, 1.03	0.4
**Education**	20				44			
High school	3	—	—		9	—	—	
Middle school	1	0.00		>0.9	1	4,471,817	0.00, NA	>0.9
Bachelor's degree	12	0.40	0.02, 11.0	0.5	26	0.33	0.04, 1.70	0.2
Master's degree or higher	4	0.67	0.02, 24.4	0.8	8	0.29	0.03, 2.17	0.2
**Marriage**	20				44			
No	4	—	—		8	0.00		>0.9
Yes	16	38,549,598	0.00, NA	>0.9	35	0.00		>0.9
Others	0				1	—	—	>0.9
**Job**	20				44			
Homemaker	6	314,366,018	0.00, NA	>0.9	18	2.36	0.31, 21.6	0.4
Office worker	5	78,591,505	0.00, NA	>0.9	8	0.90	0.09, 10.1	>0.9
Service industry worker	1	1.00	0.00, NA	>0.9	4	4.50	0.30, 138	0.3
Specialized occupation	3	1.00	0.00, Inf	>0.9	4	4.50	0.30, 138	0.3
Unskilled laborer	2	1.00	0.00, NA	>0.9	2	23,477,041	0.00, NA	>0.9
Unemployed	0				3	3.00	0.16, 97.6	0.5
Others	3	—	—		5	—	—	
**Constitution diagnostic**	20				44			
Pulmotonia	2	0.00		>0.9	2	0.00		>0.9
Colonotonia	2	4.00	0.10, 221	0.4	6	0.00		>0.9
Hepatonia	4	1.33	0.04, 44.4	0.9	10	0.00		>0.9
Cholecystonia	5	—	—		9	0.00		>0.9

	EQ-5D-5L responder versus non-responder				Symptom-specific index responder versus non-responder			
**Characteristic**	**N**	**OR***	**95% CI***	**p-value**	**N**	**OR***	**95% CI***	**p-value**
Pancreotonia	4	0.00		>0.9	9	0.00		>0.9
Renotonia	1	4.00	0.10, 221	0.4	3	0.00		>0.9
Vesicotonia	9	0.00		>0.9	11	0.00		>0.9
Unknown		—	—		2	—	—	—
Smoke	20	0.69	0.06, 16.7	0.8	44	3.50	0.87, 15.9	0.085
Drink	20	0.43	0.03, 11.0	0.5	44	1.57	0.32, 7.66	0.6
Supplementary diet	20	125,746,406	0.00, NA	>0.9	44	6.60	1.78, 27.9	**0.007**†
Baseline PainDETECT score	20	1.25	0.96, 1.76	0.13	44	1.02	0.89, 1.16	0.8
Baseline KCPAT* score**	20	1.49	1.07, 2.62	0.058	44	0.94	0.82, 1.07	0.3
Baseline EQ-5D-5L* score	20	0.00	0.00, 0.00	**0.024**†	44	0.00	0.00, 0.60	0.058

*OR = Odds Ratio, CI = Confidence Interval, KCPAT = Korean Cancer Pain Assessment Tool , EQ-5D-5L = EuroQol-5 dimension-5L

** Only the somatic pain item scores from the KCPAT were used.

†Statistically significantly different from Univariate Logistic Regression

Table S7. Analysis of covariates influencing the changes in PainDETECT and partial KCPAT scores.

	PainDETECT score change				KCPAT * score** change			
**Characteristic**	**N**	**Beta**	**95% CI***	**p-value**	**N**	**Beta**	**95% CI***	**p-value**
Sex	44				44			
Female		—	—			—	—	
Male		0.52	-3.2, 4.2	0.8		-0.24	-4.3, 3.8	>0.9
Age	44	0.11	-0.03, 0.24	0.14	44	0.00	-0.16, 0.16	>0.9
Education	44				44			
High school		—	—			—	—	
Middle school		13	2.7, 24	**0.015**†		8.6	-3.4, 20	0.2
Bachelor's degree		-0.21	-4.1, 3.7	>0.9		2.6	-1.8, 6.9	0.2
Master's degree or higher		-1.3	-6.2, 3.6	0.6		-0.82	-6.3, 4.7	0.8
Marriage	44				44			
No		-1.3	-13, 10	0.8		10	-1.5, 22	0.085
Yes		-2.7	-14, 8.2	0.6		5.2	-5.8, 16	0.3
Others		—	—			—	—	
Job	44				44			
Homemaker		-2.7	-8.1, 2.7	0.3		-7.6	-13, -1.9	**0.010**†
Unemployed		-1.3	-9.1, 6.6	0.7		-4.7	-13, 3.5	0.3
Office worker		-0.60	-6.7, 5.5	0.8		-7.6	-14, -1.2	**0.020**†
Service industry worker		-6.1	-13, 1.1	0.095		-6.5	-14, 1.0	0.087
Specialized occupation		-2.4	-9.6, 4.9	0.5		-4.5	-12, 3.0	0.2
Unskilled laborer		1.9	-7.1, 11	0.7		-4.5	-14, 4.9	0.3
Others		—	—			—	—	
Constitution diagnostic	42				42			
Pulmotonia		5.3	-2.9, 13	0.2		3.6	-5.0, 12	0.4

	PainDETECT score change				KCPAT * score** change			
**Characteristic**	**N**	**Beta**	**95% CI***	**p-value**	**N**	**Beta**	**95% CI***	**p-value**
Colonotonia		-0.22	-5.7, 5.3	>0.9		-0.22	-6.0, 5.6	>0.9
Hepatonia		-2.1	-6.9, 2.7	0.4		-4.0	-9.0, 1.1	0.12
Cholecystonia		—	—			—	—	
Pancreotonia		0.22	-4.7, 5.1	>0.9		-0.44	-5.6, 4.7	0.9
Renotonia		-6.9	-14, 0.07	**0.052**†		-9.2	-17, -1.9	**0.015**†
Vesicotonia		2.8	-4.2, 9.7	0.4		-2.2	-9.6, 5.1	0.5
Smoke	44				44			
No		—	—			—	—	
5 packs or more		1.0	-2.7, 4.7	0.6		1.2	-2.8, 5.2	0.5
Drink	44				44			
No		—	—			—	—	
Yes		-0.17	-4.4, 4.0	>0.9		-0.76	-5.3, 3.8	0.7
Supplementary diet	44				44			
No		—	—			—	—	
Yes		-1.1	-4.4, 2.2	0.5		-4.2	-7.6, -0.83	**0.016**†
Baseline PainDETECT score	44	-0.54	-0.84, -0.25	**<0.001**†	44	-0.34	-0.70, 0.02	0.062
Baseline KCPAT*score**	44	-0.48	-0.79, -0.17	**0.003**†	44	-0.59	-0.91, -0.27	**<0.001**†
Baseline EQ-5D-5L* score	44	12	-1.1, 25	0.072	44	12	-1.8, 27	0.085

*CI = Confidence Interval, KCPAT = Korean Cancer Pain Assessment Tool , EQ-5D-5L = EuroQol-5 dimension-5L

** Only the somatic pain item scores from the KCPAT were used.

†Statistically significantly different from Univariate Logistic Regression

Figure S1. Analysis of covariates influencing the changes in PainDETECT and partial KCPAT scores.

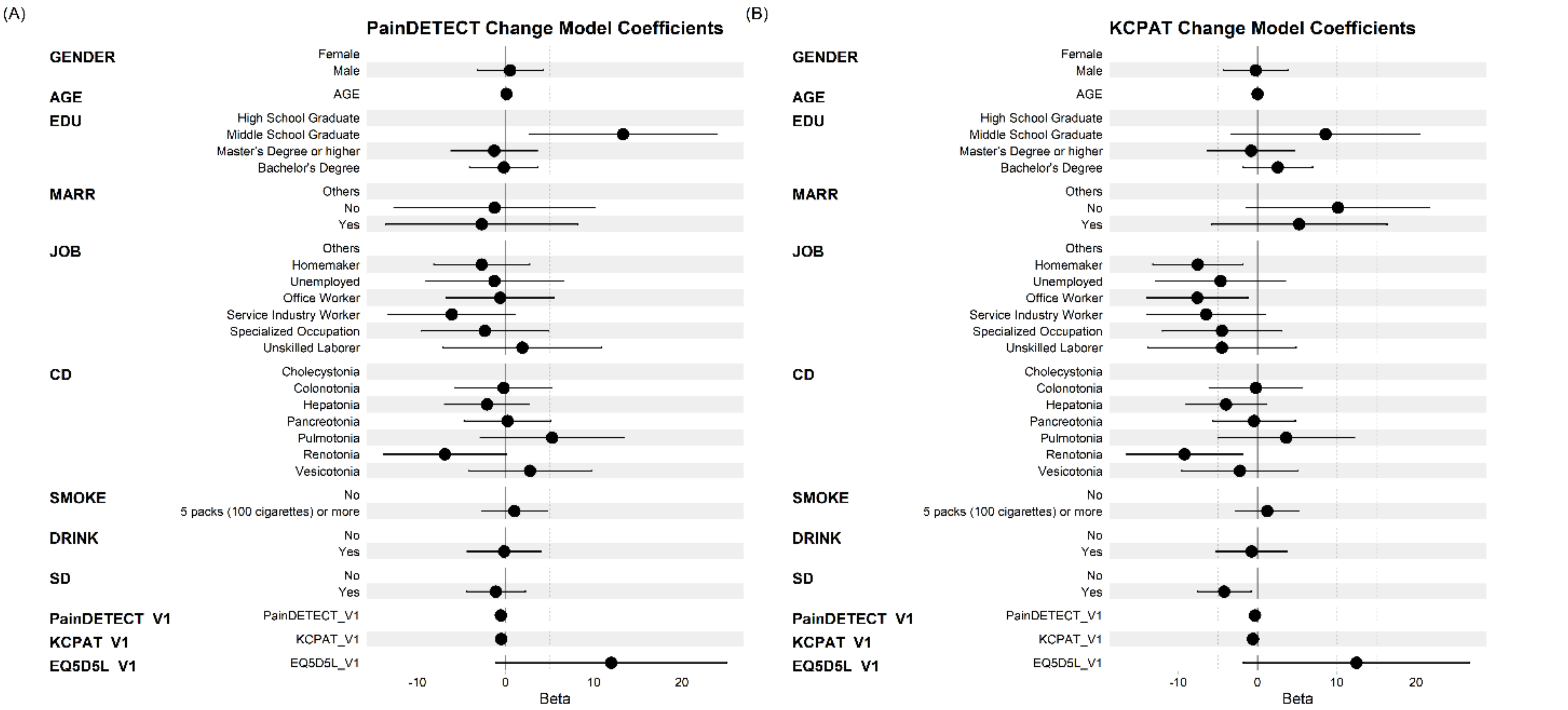

KCPAT = Korean Cancer Pain Assessment Tool, EDU=Education, MARR=marriage, CD= Constitution Diagnostic, SD= Supplementary diet, PainDETECT V1= Baseline PainDETECT score, KCPAT V1 = Baseline KCPAT score, EQ5D5L = EuroQol-5 dimension-5L

Only somatic pain item scores from the KCPAT were used.
