# Supplementary material for: Pain Treatment in Primary Care Through Eight Constitution Medicine: A Retrospective Real-World Study from South Korea
Source: Medicina (Kaunas). 2025 Aug 30;61(9):1564. doi: 10.3390/medicina61091564 (PMC12472020; doi:10.3390/medicina61091564)
Supplement: Supplementary file 1 [file medicina-61-01564-s001.zip › supplementary file 1.pdf]

Hepatonia Regimen	
When you are healthy, you sweat a lot: however, when you are weak, you do not sweat. Your body feels lighter when you sweat from various activities: that is because your constitution requires a lot of perspiration. Therefore, enjoying hot baths all the time is a good practice for your health. Mountain hiking and moderate speaking are good. Having higher blood pressure that average is a healthy state for you.	
**Harmful regimen**	**Beneficial regimen**
All kinds of ocean fishes and shellfishes, Raw cabbages and most Green and leafy Vegetables, Dextrose, Cocoa/Chocolate, Buckwheat, Bracken(fern), Persimmon, Quince, Cherry, Grapes(Wine), Swim(cold bath), Aloe vera, Dextrose injection	All kinds of Meats, Rice, Soybean, Wheat, Indian millet, Root Vegetables(radish, carrot, yam, lotus root and taro), Coffee, Milk(warm), Garlic, Pumpkin or squash, Mushrooms, Sugar, Some fresh water fishes(eel, loach/mudfish), Alkaline beverages, Pear, Apple, Watermelon, Nuts(walnut, pecan, chestnut, pine-nut), Deer horn(herb), Ginseng, Vitamin A, B and D

This is the regimen based on 8 Constitution Medicine by Dr. Dowon Kuon

Cholecystonia Regimen	
Discomfort in your lower abdomen is large intestinal inertia, which is the very cause of such symptoms as a heavy feeling in your legs, backache, irregular bowl movements, depression, cold body and occasional insomnia. Therefore, it is good to keep your lower abdomen warm all the time. As your constitution is prone to becoming alcoholic, it is necessary to take extra precautions in consuming alcohol.	
**Harmful regimen**	**Beneficial regimen**
All kinds of ocean fishes and shellfishes, Alcoholic beverages, Raw cabbages and most Green and Leafy Vegetables, Buckwheat, Bracken(fern), Cocoa/Chocolate, Grapes(Wine), Cherry, Persimmon, Quince, Dextrose, Ginseng, Swim(cold bath)	Beef, Pork, Rice, Soybean, Wheat, Indian millet, Root Vegetables(radish, carrot, yam, lotus root and taro), Coffee, Milk(warm), Garlic, Pumpkin or squash, Mushrooms, Sugar, All kinds of nuts(walnut, pecan, chestnut, pine-nut), Some fresh water fishes(eel, loach/mudfish), Alkaline beverages, Pear, Melons, Deer horn(herb), Squalene oil, Vitamin A and D

This is the regimen based on 8 Constitution Medicine by Dr. Dowon Kuon

Pancreotonia Regimen	
Your health is so directly related to your tendency to be quick-tempered that your health practice should always be to keep your poise and not to hurry. Although your constitution has strong digestive powers, you should avoid food that doesn't suit your constitution. Alcohol and cold baths are detrimental.	
**Harmful Regimen**	**Beneficial Regimen**
Chicken and Poultry, Lamb, Brown rice, Seaweeds, Apple, Orange/Citrus, Mango, Ginseng, Potato, Honey, Vitamin B group, Pepper, Ginger, Green onion, Onions, Mustard, Cinnamon, Curry, Hot & Spices, Sesame oil, Dates, Digestive enzyme, Antibiotics, Cold bath	Barley, Rice, Eggs, Wheat, Beans and nuts, Red beans, Pork, Beef, Green Vegetables, All kinds of fishes and shellfishes, Persimmon, Pear, Melons, Strawberry, Cranberry and most Berries, Banana, Vitamin E, Ice, Aloe vera, Mushrooms

This is the regimen based on 8 Constitution Medicine by Dr. Dowon Kuon

Gastrotonia Regimen	
You have to be careful with medications and foods since this constitution is prone to indigestion due to side effects from them. Cool, fresh food is beneficial while alcohol and cold baths are detrimental.	
**Harmful Regimen**	**Beneficial Regimen**
Sweet/Brown rice, Chicken and Poultry, Lamb, Mustard, Red and black pepper, Cinnamon, Curry, Ginger, Green onion, Hot & Spices, Apple, Orange/Citrus, Mango, Tomato, Seaweed, Burnt foods, Ginseng, Dates, Honey, Vitamin B group, Penicillin, Alcohol, Smoking cigarette	Barley, Rice, Red beans, Mung beans, Cucumber, Green Vegetables, All kinds of ocean fishes and shellfishes, Swellfish, Pork, Beef, Persimmon, Melons, Pineapple, Grapes, Strawberry, Banana, Aloe vera, Ice, Chocolate, Vitamin E

This is the regimen based on 8 Constitution Medicine by Dr. Dowon Kuon

GSK ECM AcuClinic

<table>
<tr><th colspan="2">Pulmotonia Regimen</th></tr>
<tr><td colspan="2">The reason why medication is as effective as it is harmful and your body suffers after eating meat is because your liver is functioning weakly. Therefore, your healthy foods should be vegetables and seafood. The secret to your health is spending a lot of time standing with your back straight. Avoid sunbathing and excessive perspiration.</td></tr>
<tr><th>Harmful Regimen</th><th>Beneficial Regimen</th></tr>
<tr><td>All kinds of Meats, Milk, All fresh water fishes, Coffee/Tea, Artificial seasonings/drinks, Wheat flour, Indian millet, Pumpkin or squash, Peppers, Garlic, Mushrooms, Sugar, Root Vegetables, Soy bean, All kinds of Nuts, Apples, Pear, Deer horn(herb), Ginseng, All medicinal substances, Vitamin A, B and D, Alkaline beverages, Alcohol and Cigarette, Hot Bath(Sauna)</td><td>All kinds of ocean fishes and shellfishes, Rice(White), Buckwheat, Mung beans, Mugwort, Cucumber, Eggplant, Cabbage, Lettuce, Green Vegetables, Bracken(fern), Dextrose, Cocoa/Chocolate, Banana, Strawberry, Peach, Cherry, Persimmon, Quince, Aloe vera, Ice, Dextrose injection</td></tr>
</table>

This is the regimen based on 8 Constitution Medicine by Dr. Dowon Kuon

Colonotonia Regimen	
The first health principle for this constitution is to stop eating any kind of meat and the second is not to use medication. The third is not to get upset. In case you have muscle inertia symptoms, extra precaution is required and it's beneficial to take cold baths regularly.	
**Harmful Regimen**	**Beneficial Regimen**
All kinds of Meats, Milk, Garlic, Deer horn(herb), All fresh water fishes, Artificial seasonings, Wheat flour, Indian millet, Pumpkin or squash, Soy bean, Sugar, Pear, Apples, Melons, Chestnut, Pine-nut, Ginkgo, All kinds of Nuts, Root Vegetables, Mushrooms, Vitamin A, D and E, Alkaline beverages, Hot Bath(Sauna)	Buckwheat, Rice(White), Dextrose, All kinds of ocean fishes and shellfishes(except oyster), Green Vegetables, Cucumber, Bracken(fern), Green seaweed, Grapes, Peach, Persimmon, Cherry, Pineapple, Strawberry, Mustard, Cocoa/Chocolate, Acanthopanax root bark, Swim(cold bath)

This is the regimen based on 8 Constitution Medicine by Dr. Dowon Kuon

<table>
<tr><th colspan="2">Renotonia Regimen</th></tr>
<tr><td colspan="2">The reason you are healthier in the colder seasons is because you should not sweat a lot constitutionally. Therefore, the only healthy way to avoid perspiration is to enjoy cold baths or rubbing your body with a cold towel.</td></tr>
<tr><th>Harmful Regimen</th><th>Beneficial Regimen</th></tr>
<tr><td>Barley, Red beans, Cucumber, Pork, Oyster and shellfishes, Persimmon, Melons, Strawberry, Banana, Pineapple, Beer, Ice, Vitamin E, Cranberry, Aloe vera, Mushrooms, Mercury, Hot Bath(Sauna)</td><td>Sweet/Brown rice, Chicken and Poultry, Lamb, Beef, Seaweeds, Cinnamon, Ginger, Green onion, Mustard, Red and black pepper, Sesame oil, Potato, Apple, Mango, Orange/Citrus, Tomato, Ginseng, Honey, Dates, Vitamin B group</td></tr>
</table>

This is the regimen based on 8 Constitution Medicine by Dr. Dowon Kuon

Vesicotonia Regimen	
If you have food cold in temperature as well as quality, your cold stomach will become colder and lead you to unhealthy and anxious states, eventually to gastroptosis. Therefore, your first health principle should be light eating and eating warm food.	
**Harmful Regimen**	**Beneficial Regimen**
Barley, Red beans, Cucumber, Pork, Eggs, Swellfish, All kinds of shellfishes, Persimmon, Melons, Banana, Strawberry, Grapes(Wine), Beer, Chocolate, Aloe vera, Ice, All types of cold beverages and foods, Vitamin E, Alkaline Beverages, Mecury, Smoking cigarette, Hot bath(Sauna)	Sweet/Brown rice, Potato, Corn, Sesame oil, Seaweeds, Chicken and Poultry, Lamb, Red and black pepper, Mustard, Cinnamon, Curry, Green onion, Ginger, Apple, Orange/Citrus, Tomato, Mango, Ginseng, Dates, Honey, Vitamin B group

This is the regimen based on 8 Constitution Medicine by Dr. Dowon Kuon
